# UGT1A1 gene variations and irinotecan treatment in patients with metastatic colorectal cancer

**DOI:** 10.1038/sj.bjc.6602042

**Published:** 2004-07-27

**Authors:** E Marcuello, A Altés, A Menoyo, E del Rio, M Gómez-Pardo, M Baiget

**Affiliations:** 1 Department of Medical Oncology, Hospital de la Santa Creu i Sant Pau. Avda. S. Antoni M Claret 167, 08025 Barcelona, Spain; 2 Department of Hematology, Hospital de l’Esperit Sant. Avda. M. Josep Pons i Rabada s/n, 08923 Sta. Coloma de Gramanet, Barcelona, Spain; 3 Department of Genetics, Hospital de la Santa Creu i Sant Pau. Avda. S. Antoni M^a^ Claret 167, 08025 Barcelona, Spain

**Keywords:** irinotecan, SN-38, UGT1A1, pharmacogenetics

## Abstract

SN-38 is the active metabolite of irinotecan and it is metabolised through conjugation by uridine diphosphate glucuronosyl transferase (UGT1A1). The major toxicity of irinotecan therapy is diarrhoea, which has been related to the enzymatic activity of UGT1A1. We examined the influence of the UGT1A1 gene promoter polymorphism in the toxicity profile, in the response rate and in the overall survival (OS) in 95 patients with metastatic colorectal cancer treated with an irinotecan-containing chemotherapy. Genotypes were determined by analysing the sequence of TATA box of UGT1A1 of genomic DNA from the patients. Clinical parameters and genotypes were compared by univariate and multivariate statistical methods. The more frequent adverse effects were asthenia (34 patients), diarrhoea (29 patients) and neutropenia (20 patients). Severe diarrhoea was observed in 7/10 homozygous (70%) and 15/45 heterozygous (33%) in comparison to 7/40 (17%) wild-type patients (*P*=0.005). These results maintained the statistical significance in logistic regression analysis (*P*=0.01) after adjustment for other clinical relevant variables. The presence of severe haematological toxicity increased from wild-type patients to UGT1A1^*^28 homozygotes, but without achieving statistical significance. No relationship was found between the UGT1A1^*^28 genotypes and infection, nausea or mucositis. In univariate studies, patients with the UGT1A1^*^28 polymorphism showed a trend to a poorer OS (*P*=0.09). In the multivariate analysis, the genotype was not related to clinical response or to OS. The role of the UGT1A1 genotype as a predictor of toxicity in cancer patients receiving irinotecan demands the performance of a randomized trial to ascertain whether genotype-adjusted dosages of the drug can help to establish safe and effective doses not only for patients with the UGT1A1^*^28 homozygous genotype but also for those with the most common UGT1A1 6/6 or 6/7 genotype.

Irinotecan (CPT-11) is currently used in cancer chemotherapy given its ability to inhibit topoisomerase I (Jaxel *et al*, 1989; Bissery *et al*, 1996; Zamboni *et al*, 1998). On the basis of large randomised clinical trials, irinotecan either alone or in combination with fluorouracil has been accepted as first- or second-line chemotherapy for the treatment of patients with colorectal cancer (Cunningham *et al*, 1998; Rougier *et al*, 1998; Douillard *et al*, 2000; Saltz *et al*, 2000). The most common side effects are bone marrow toxicity, leading to abnormally low blood counts, and ileocolitis, which often results in severe diarrhoea (Vanhoefer *et al*, 2001). These adverse effects impair the therapeutic efficacy, and could result in the discontinuation of an otherwise effective anticancer treatment.

Irinotecan is mainly eliminated unchanged by the liver and to a minor extent by the kidneys (Mathijssen *et al*, 2001). The drug follows two metabolic pathways that take place in the liver. It can be converted into an inactive metabolite by the CYP3A4 cytochrome (Haaz *et al*, 1998) and into an active metabolite, SN-38, by carboxylesterase enzymes (Slatter *et al*, 1997). SN-38 is further metabolised through conjugation into SN-38 glucuronide (SN-38G) by uridine diphosphate glucuronosyltransferase (UGT1A1), the same enzyme that conjugates bilirubin (Iyer *et al*, 1998).

In a study performed to investigate the effect of glucuronidation on the concentration of SN-38 following irinotecan infusion in 21 patients undergoing a phase I trial, an inverse relationship between SN-38 glucuronidation rates and severity of diarrhoea has been reported, indicating that glucuronidation of SN-38 might protect against irinotecan-induced gastrointestinal toxicity (Gupta *et al*, 1994). An interesting case-report on two patients with metastatic colorectal cancer and Gilbert's syndrome (a chronic nonhaemolytic unconjugated hyperbilirrubinaemia caused by a reduction in the activity of UGT1A1) treated with CPT-11 provided the first clinical evidence linking deficiency in the UGT1A1 activity and irinotecan-related toxicity (Wasserman *et al*, 1997).

The UGT1A locus in humans is located in the long arm of chromosome 2 (2q37) and spans approximately 160 kb. The UGT1A1 gene consists of at least nine promoters and first exons (first exons 3, 11 and 12 are pseudogenes) that can be spliced with four common exons to result in nine different UGT1A1 enzymes (Ritter *et al*, 1992; Owens and Ritter, 1995). More than 50 genetic variations in the promoter and coding regions of the gene are currently known to decrease the enzyme activity (Kadakol *et al*, 2000), leading to constitutional unconjugated jaundice (Crigler-Najjar or Gilbert's syndromes). One of the most common genotypes causing Gilbert's syndrome in Caucasian populations is the inheritance of a promoter region containing an extra TA dinucleotide [A(TA)_7_TAA], which results in a 70% reduction in transcriptional activity compared with wild-type UGT1A1 [A(TA)_6_TAA]. Patients who are either heterozygous or homozygous for this variant allele (designated as UGT1A1^*^28) exhibit an attenuated expression of UGT1A1 and are theoretically predisposed to SN-38 initiated diarrhoea (Ando *et al*, 2000). Furthermore, in a human liver microsome experimental model, a significant trend towards a decrease in SN-38 and bilirrubin glucuronidation rates was found as the number of TA dinucleotide repeats increased (6/6>6/7>7/7) (Iyer *et al*, 1999).

In the present study, we examined the influence of uridine diphosphate glucuronosyl transferase UGT1A1 polymorphism on the toxicity profile, on the response rate and on the overall survival (OS) in patients with metastatic colorectal cancer treated with an irinotecan-containing chemotherapy.

## MATERIALS AND METHODS

### Patients

In all, 95 patients diagnosed with metastatic colorectal cancer and undergoing irinotecan-based chemotherapy were studied. All patients were primarily ensured to have an adequate bone marrow and organ function before the use of irinotecan. The exclusion criteria were ECOG ⩾3 and apparent jaundice. All patients gave written informed consent, and the study was approved by the Institutional Ethics Committee.

### Chemotherapy regimen description

We used four different regimens in this group of patients. Regimen A consisted of irinotecan alone (350 mg m^2^ infused in 45 min i.v. every 3 weeks). Regimen B consisted of irinotecan at the same dose and intervals with Tomudex (3 mg m^2^ infused in 15 min i.v. in every cycle). Regimen C consisted of irinotecan (80 mg m^2^ infused in 45 min i.v. every week) with a dose of 2250 mg m^2^ of 5-FU (in continuous infusion during 48 h i.v.) in every cycle. Regimen D consisted of irinotecan (180 mg m^2^ every 2 weeks i.v.) and 5-FU with leucovorin i.v. Patients underwent chemotherapy cycles until severe toxicity or disease progression (DP) appeared.

### Clinical parameters

Relevant clinical data were obtained from clinical records (gender, age, ECOG, previous surgery or radiotherapy, line of chemotherapy, actual dosage of irinotecan, and use of G-CSF). Two serum total bilirubin levels were analysed: just prior to irinotecan administration and the highest value after initiation of therapy. Toxicity was graded in accordance with the WHO scale: presence and grade of nausea, asthenia, mucositis, diarrhoea, infection, neutropenia, anaemia and thrombocytopenia. Response to treatment and OS were also analysed. Complete remission (CR) was defined as the disappearance of tumor masses and disease-related symptoms, as well as the normalisation of the initially abnormal tests and/or biopsies lasting for at least 1 month. Partial remission (PR) was considered when measurable lesions decreased by at least 50%. Clinical response was assumed when a complete or PR was obtained. Patients without criteria of clinical response but without progression were considered patients with stable disease (SD). Disease progression during or after treatment was also considered. Overall survival was calculated from the start of chemotherapy to death regardless of the cause.

**Figure 1:**
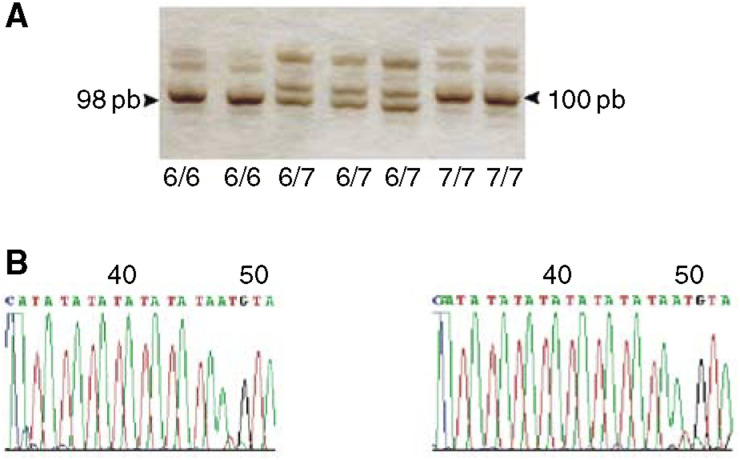
Analysis of the A(TA)nTAA motif, in the promoter region of the UGT1A1 gene. Pattern of polyacrylamide gel electrophoresis: DNA bands of 98 bp correspond to alleles containing six TA repeats. DNA bands of 100 pb correspond to alleles containing seven TA repeats. Automated sequencing of the DNA fragments with six and seven TA repeats.

UGT1A1 genotyping assay: Genomic DNA was extracted from peripheral leukocytes by the salting-out procedure (Miller *et al*, 1989). Analysis of the A(TA)nTAA motif in the promoter region of the UGT1A1 gene was performed by PCR, according to Monaghan *et al* (1996), followed by separation of the amplified products on a 12% polyacrylamide gel (38 : 2 acrylamide : bisacrylamide). Following this protocol, DNA fragments containing six TA repeats measure 98 bp while those containing seven TA repeats occur as 100 bp bands. Automated sequencing was performed to confirm these sizes (Figure 1

).

### Statistical analysis

Differences between categorical variables were measured by the *χ*^2^ test. Differences between means in continuous variables were measured by: the Mann–Whitney *U* test when the variables were two and unrelated, the Wilcoxon signed-rank test when the variables were two and related, and analysis of variance when we had more than two unrelated variables. Logistic regression was used as a multivariate method to ascertain whether the UGT1A1 genotype independently predicted toxicities significantly related to this variable in previous univariate analysis. Kaplan–Meier estimates and the log-rank test were the methods used in univariate analysis of OS. A Cox-regression model was used for OS multivariate analysis. The results were considered as statistically significant when bilateral *P*-values were less than 0.05.

## RESULTS

### Clinical and chemotherapy data and adverse effects

A total of 95 patients who fulfilled all inclusion and exclusion criteria were studied. Clinical data, chemotherapy characteristics and response to treatment are shown in Table 1

**Table 1:** Baseline characteristics of the 74 patients, chemotherapy and response to treatment.

Gender (men/women)	60 (63%)/35 (37%)
Median age (range, year)	68 (25–83)
*Performance status*	
0	46 (48%)
1	29 (31%)
2	0 (21%)
	
*Previous treatment*	
Surgery	93 (98%)
Radiotherapy	11 (12%)
	
*Chemotherapy treatment*	
CPT-11 alone	12 (13%)
CPT-11 and Tomudex	9 (9%)
CPT-11 and 5FU	18 (19%)
CPT-11 and 5FU+LV	56 (59%)
	
*Line of chemotherapy*	
First line	57 (60%)
⩾Second line	38 (40%)
	
Mean total actual dosage mg m^−2^ (range)	1660 (180–6300)
	
*Use of G-CSF*	
Yes	10 (11%)
Not	85 (89%)
	
*Objective response*	
Complete or partial	26 (27%)
Stable disease	32 (34%)
Progressive disease	22 (23%)
Not evaluable	15 (16%)

. Severe toxicities (Grades III–IV) were frequent in this group of patients. In total, 55% (58%) suffered from some kind of grade III/IV adverse effect and 28 out of these patients (48%) developed more than two severe adverse effects at the same time. The most frequent severe adverse effects were asthenia (34 patients), diarrhoea (29 patients) and neutropenia (20 patients).

### UGT1A1 genotype and bilirubin metabolism

The total serum bilirubin level prior to therapy and the highest level during the chemotherapy cycles were measured and were related to the UGT1A1 genotype. The results are shown in Table 2

**Table 2:** UGT1A1^*^28 genotypes and bilirubin levels (*μ*mol l^−1^) prior to irinotecan administration and the highest value during therapy

**Genotype^a^**	**N**	**Bilirubin premean (range)**	**Bilirubin higher mean (range)**	P*
6/6	40	8.3 (4–22)	9.9 (4–25)	0.001
6/7	45	8.7 (4–18)	13 (5–37)	0.001
7/7	10	15 (6–28)	22 (6–65)	0.1
*P*	—	0.001	0.001	

a Symbols of (6/6), (6/7) and (7/7) denote homozygous absence of the variant allele, heterozygous, and homozygous for the variant allele, respectively. P=Significance of comparisons of bilirubin levels between genotypes. P^*^=Significance of comparisons of bilirubin levels pre- and postchemotherapy.

. Differences in the mean of bilirubin levels of the three genotypes were significant pre- and postchemotherapy. Bilirubin levels increased significantly when chemotherapy was initiated in 6/6 patients (*P*=0.01) and in 6/7 patients (*P*=0.001); in the group of 7/7 patients, this increase, although very marked, did not attain significance (*P*=0.1) because of the small number of cases.

### UGT1A1 genotype and toxicity

The allelic frequency of UGT1A1^*^28 in the group of patients analysed was 0.34 (IC95%, 0.28–0.41), within the range reported in Caucasian populations. Table 3

**Table 3:** Associations between UGT1A1^*^28 genotypes and grade III–IV toxicities

**Genotype^a^**	**N**	**Asthenia**	**Diarrhoea**	**Haematological**	**Nausea**	**Mucositis**	**Infection**
**6/6**	40	10 (25%)	7 (17%)	6 (15%)	5(12%)	1 (3%)	6 (15%)
**6/7**	45	17 (38%)	15 (33%)	12 (27%)	10 (22%)	2 (4%)	2 (4%)
**7/7**	10	7 (70%)	7 (70%)	4 (40%)	2 (20%)	0 (0%)	0 (0%)
***P***	**—**	**0.03**	**0.005**	0.2	0.4	0.4	0.13

a Symbols of (6/6), (6/7), and (7/7) denote homozygous absence of the variant allele, heterozygous and homozygous for the variant allele, respectively. Bold values refer to statistically significant values.

shows the univariate relationships between the UGT1A1 genotypes and severe adverse effects (grades III and IV). There was a marked relationship between the appearance of severe diarrhoea (*P*=0.005) and asthenia (*P*=0.03) and the heterozygous and homozygous UGT1A1^*^28 condition. These two variables were clearly related: 53% of patients with severe diarrhoea also had severe asthenia, whereas 82% of the patients who did not develop severe asthenia did not have diarrhoea either (*P*=0.001). In these cases, it may be assumed that the variable related to the genotype was diarrhoea causing asthenia. The presence of severe haematological toxicity (grades III/IV neutropenia, anaemia or thrombocitopenia) increased from wild-type patients to UGT1A1^*^28 homozygotes, but did not achieve statistical significance. No relationship was found between the UGT1A1^*^28 genotypes and infection, nausea or mucositis.

We have compared the preteatment levels of serum bilirrubin between the group of 29 patients with grades III–IV diarrhea (9.6–5.5 *μ*mol l^−1^) and the group of 65 patients without this adverse effect (9.1–3.7 *μ*mol l^−1^). No significant differences were observed. (*P*=0.5).

A logistic regression model was constructed to ascertain whether UGT1A1^*^28 genotype was independently related to the appearance of severe diarrhoea. Table 4

**Table 4:** Multiple logistic regression analysis for variables independent and significantly related to the presence of severe diarrhoea

**Term**	***β***	**s.e.**	***χ^2^***	***P***	**Odds ratio (95% CI)**
Intercept	−1.5	0.5			
UGT1A1			9.0	0.01	
UGT1A1 +/−	0.9	0.5	3	0.1	2.4 (0.9–6.6)
UGT1A1 +/+	2.4	0.8	8.8	0.03	11 (2.3–5.3)

shows the results of this multivariate model. The UGT1A1 genotype was the only variable that significantly predicted the appearance of severe diarrhoea. Other variables included in the model but with no statistical significance were age, gender, performance status, pretreatment levels of serum bilirrubin, previous surgery or radiotherapy, chemotherapy regimen and lines of chemotherapy.

### UGT1A1^*^28 genotypes, efficacy of treatment and OS

**Figure 2:**
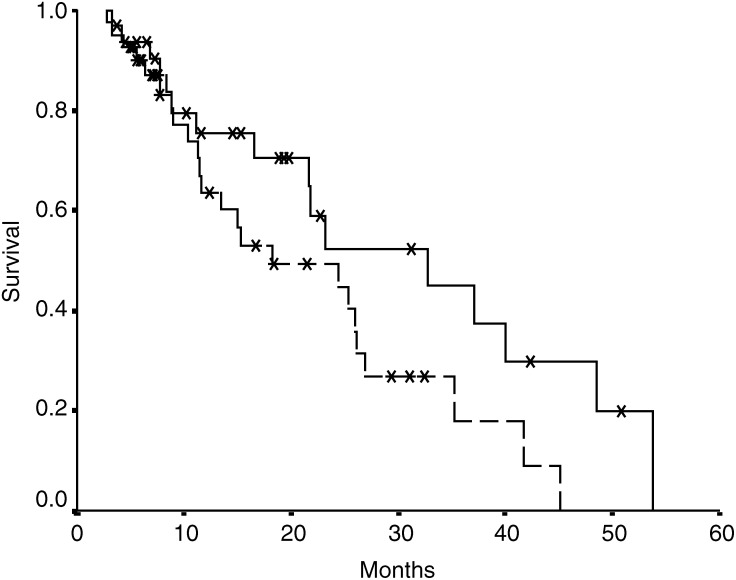
Overall survival of patients with ‘wild-type’ UGT1A1 (continuous line) and patients heterozygous and homozygous for UGT1A1^*^28 (dotted line); *P*=0.07.

Although in univariate studies, patients with the UGT1A1^*^28 polymorphism did not have a statistically different probability of achieving a clinical response (*P*=0.3), they showed a trend to a poorer OS (median OS 33 months for 6/6 patients *vs* 21 months for 6/7 and 7/7 patients, *P*=0.09) (Figure 2

). In the multivariate analysis no genotype was related to clinical response or to OS (*P*=0.1 and 0.8, respectively).

## DISCUSSION

Although more than 50 genetic lesions in the UGT1A1 gene have been described (Kadakol *et al*, 2000), the UGT1A1^*^28 allele (the most frequent polymorphism in Caucasian populations) plays a crucial role in the development of toxicity after irinotecan chemotherapy.

In a preliminary study with nine patients, Ando *et al* (1998) observed a lower glucuronidation rate of SN-38 in a patient with genotype 7/7 *vs* those with genotypes 6/7 (*n*=1) and 6/6 (*n*=7), suggesting that the patient with genotype 7/7 had an impaired capacity for glucuronidation of SN-38.

In a subsequent work, these authors observed a heightened sensitivity to irinotecan induced toxicity in a retrospective and case-controlled study with 118 Japanese patients with different cancers (Ando *et al*, 2000). They reported a frequency of the UGT1A1^*^28 allele 3.5-fold higher in patients with toxicity (severe diarrhoea or leukopenia) compared with patients without this complication (*P*<0.0001). Multivariate analysis indicated that the presence of the UGT1A1^*^28 allele turned out to be a risk factor for severe toxicity (*P*<0.001; odds ratio, 7.23; 95% confidence interval, 2.52–22.3). A trend to a better response of patients who experienced toxicity was observed, but this had no statistical significance. The authors suggested that the determination of the UGT1A1 genotypes might be clinically useful for predicting severe toxicity by irinotecan in cancer patients.

The pharmacogenetic assessment of UGT1A1 polymorphism was also investigated in 20 patients with several primary malignancies who were treated with irinotecan at a dose of 300 mg m^2^ every 3 weeks (Iyer *et al*, 2002). Circulating levels of SN-38 were higher in those patients harbouring the TA7 allele. The time course of the ratio of plasma concentration of SN-38G to SN-38 indicated much lower glucuronidation rates of SN-38 for patients with genotypes 6/7 and 7/7 when compared with those that were homozygous 6/6 at each blood sampling time. There was a significant progressive reduction in SN-38 glucuronidation rates and a significant increase in AUC SN-38 values in patients with genotype 6/7 and 7/7. All the patients with two wild-type alleles had no/low diarrhoea or mild leukopenia. Severe grades of diarrhoea and neutropenia were scored only in patients with genotypes 6/7 and 7/7. However, the differences were not statistically significant for neutropenia or diarrhoea grades among the three genotypes.

In a recent work (Mathijssen *et al*, 2003), an exhaustive genotype analysis of the irinotecan pathway was carried out in 65 cancer patients treated with irinotecan (200–350 mg m^2^ in an i.v. 90-min infusion). The extent of SN-38 glucuronidation was slightly impaired in homozygous variants of UGT1A1^*^28, although differences were not statistically significant given the very low frequency of the variant allele in this group of patients. Only two patients were UGT1A1^*^28 homozygotes and one of them was the only patient in the entire cohort to suffer from grade IV diarrhoea.

In the present work, which includes 95 patients with metastatic colorectal cancer, a statistically significant relationship, in both univariate and multivariate analyses, was found between the appearance of severe diarrhoea and the homozygote UGT1A1^*^28 genotype when compared with UGT1A1 wild-type patients. We found no relationship between haematological toxicity and genotype.

To our knowledge, this is the first pharmacogenetic study carried out exclusively on patients with colorectal cancer. In our series, 98% of patients had been submitted to surgery and 12% to local radiotherapy. This could account for both the high frequency of severe diarrhoea observed and the statistically significant association of this adverse effect with the UGT1A1 genotype.

Levels of bilirubin pre- and postchemotherapy were different for each genotype (6/6, 6/7 and 7/7). Median bilirubin levels increased significantly with treatment in 6/6 and 6/7 patients. Although the increase was particularly marked in 7/7 patients (7 *μ*mol l^−1^), statistical significance was not achieved because of the small number of patients (*n*: 10).

No improvement was observed in the clinical responses in carriers and/or homozygous cases of UGT1A1^*^28, but a trend to a better survival (*P*=0.09) was found in ‘wild-type’ patients in univariate analysis. These findings could be the result of a reduction in the dosing of irinotecan in UGT1A1^*^28 carriers and/or homozygous (because of the appearance of severe diarrhoea). In fact, doses of CPT-11 administered in each genotypic group were 1725 mg m^2^ in 6/6, 1659 mg m^2^ in 6/7 and 1398 mg m^2^ in 7/7. These doses have a decreasing tendency although the differences were not significant (*P*=0.2).

Anticancer agents are usually administered in accordance with the body-surface area, but in the case of irinotecan, this parameter does not have a clinically meaningful correlation with any pharmacokinetic parameter. The UGT1A1 genotype would therefore be more useful than the body-surface area in the pharmacokinetics of irinotecan (Mathijssen *et al*, 2002).

The available information regarding the role of the UGT1A1 genotype as a predictor of toxicity in cancer patients receiving irinotecan demands the performance of a randomized trial to ascertain whether genotype-adjusted dosages of the drug can help to establish safe and effective doses not only for patients with the UGT1A1^*^28 homozygous genotype but also for those with the most common UGT1A1 6/6 or 6/7 genotype. Irinotecan studies have paved the way for new genetic-based ways of applying chemotherapeutic treatments in a more rational manner.
